# AMCD: A multi-domain agricultural crop and flower image dataset for deep learning-based classification

**DOI:** 10.1016/j.dib.2026.112974

**Published:** 2026-06-13

**Authors:** Md Ahsan Karim, Md Tanjum An Tashrif, Shahariar Hossain Mahir, Md Kowsar Ahmed, Umme Sara, Mohammad Shorif Uddin, Tufayel Haque Raha

**Affiliations:** a Department of Computer Science and Engineering, National Institute of Textile Engineering and Research (NITER), Nayarhat, Savar, Dhaka 1340, Bangladesh; b Department of Computer Science and Engineering, Jahangirnagar University, Savar, Dhaka 1342, Bangladesh

**Keywords:** Agricultural image dataset, Crop classification, Transfer learning, Computer vision, Agricultural AI, Field-condition imagery, Bangladesh agriculture, Multi-class classification

## Abstract

The Agricultural Multidisciplinary Collection Dataset (AMCD) contains 5405 JPG images of agricultural crops and flowers collected in Bangladesh. The images are organized into four domains: fruits, vegetables, flowers, and crops/grains. These domains contain 77 subclasses representing commonly observed agricultural and floricultural specimens. Images were captured manually using smartphone cameras at farms, marketplaces, and gardens in Savar, Dhamrai, and Manikganj in the Dhaka Division of Bangladesh between 27 January 2025 and 20 May 2025. The data include natural outdoor lighting, variable backgrounds, different viewpoints, single-object scenes, and multi-object scenes. After collection, images were cleaned, resized to 512 × 512 pixels, color balanced, contrast enhanced, and edge sharpened. Conservative non-synthetic augmentation was applied to underrepresented subclasses using horizontal flipping, small rotations, and brightness adjustment. The dataset can be reused for agricultural image classification, transfer learning, model benchmarking, lightweight mobile model development, domain adaptation, and healthy-specimen reference data in plant image analysis.

Specifications Table

**Table:** 

Subject	Computer Sciences
Specific subject area	Agricultural AI, image classification, deep learning, computer vision, precision agriculture
Type of data	Image (.JPG)
Data collection	The images were photographed manually using a Samsung Galaxy A21s (48MP) and an iPhone 12 Pro Max (12MP) on various farms, marketplace venues, and gardens of the Savar, Dhamrai, and Manikganj regions of Bangladesh
Data source location	City/Town/Region: Savar, Dhamrai, and Manikganj, Dhaka Division. Country: Bangladesh.
Data accessibility	Repository name: Mendeley Data Data identification number: 10.17632/7rv9wg3ksd.2 Direct URL to data: https://data.mendeley.com/datasets/7rv9wg3ksd/2 Instructions for accessing these data: The dataset is publicly available through the repository URL and DOI.
Related research article	None

## 1 Value of the Data

• These data provide 5405 agricultural and flower images captured under field and marketplace conditions in Bangladesh, covering fruits, vegetables, flowers, and crops/grains.
• The 77 subclasses support multi-class image classification and can be used to compare convolutional neural networks, deep learning models, transfer learning, and image classification task.
• The images include natural variation in illumination, background, viewpoint, object scale, and object framing, which supports robustness testing for agricultural computer vision models.
• The dataset can be reused to develop mobile and low-resource agricultural applications, including crop recognition and visual sorting. With appropriate metadata indexing, it may also support agricultural image retrieval systems in future work.
• The healthy-specimen images can be reused as reference or negative-control data in studies that compare healthy and diseased plant or crop imagery.
• The standardized 512 × 512 pixels JPG format and organized subclass structure make the data reusable for benchmarking, transfer learning, domain adaptation, and cross-dataset generalization studies.

## 2 Background

Applications in agricultural computer vision use visual and/or video data to perform tasks such as crop recognition, detecting crop diseases, and implementing precision agriculture. Agricultural computer vision has been used in crop recognition, disease detection, and precision agriculture applications [1,2]. In recent years, there has been excitement in the fields of convolutional neural networks (CNNs), lightweight models, and transfer learning, resulting in increased demand for diverse collections of field collected data used to facilitate supervised learning [3,4]. However, most publicly available datasets are narrow in scope, focusing solely on certain crops, disease types, post-harvest evaluations, or evaluations in controlled environments [[5], [6], [7]]. Virtually no datasets consist of more than a small number of agricultural categories under real-world farming conditions (e.g., fruits, vegetables, flowers, and grains). Furthermore, most datasets contain only a limited number of crops and fruit varieties in a region or only crop disease images for a particular plant species [6,7]. This research aims to provide agricultural researchers a comprehensive image dataset containing 5405 images collected in the field of agriculture, across 77 subclasses of four primary agricultural categories (fruits, vegetables, flowers, or grains) to address this limitation. This dataset consists of smartphone images taken under various conditions (e.g., natural light; varied background, viewpoint, and object size) that can support agricultural image classification, transfer learning, semantic segmentation, and comparative analysis between classification and segmentation algorithms under real world conditions [[8], [9], [10]]. Recent advances in smart agriculture have demonstrated that image datasets such as AMCD can serve as foundational components in broader IoT-assisted farming systems. It can support tasks such as pest forecasting, crop monitoring, and resource optimization [11]. The healthy-specimen images in this dataset are intended to serve as a necessary baseline and negative control reference for future disease detection research. Without such a baseline, distinguishing healthy from diseased tissue in comparative studies lacks a standardized reference point.

## 3 Data Description

The Agricultural Multidisciplinary Collection Dataset (AMCD) contains 5405 field collected images distributed across four agricultural domains: Fruits, Vegetables, Flowers, and Farming/Cereals. These images cover 77 subclasses captured under natural outdoor conditions at different farm locations throughout Bangladesh. Most subclasses contain around 70 images after preprocessing and non-synthetic augmentation, while the Dahlia flower subclass has the highest number of images (85) due to the availability of additional images.

All images are stored in JPG format with a uniform resolution of 512 × 512 pixels to maintain consistency in size and shape. The dataset includes both single object and multi object scenes with natural variation in lighting, background complexity, viewpoint, and object scale.

**Fig. 1:**
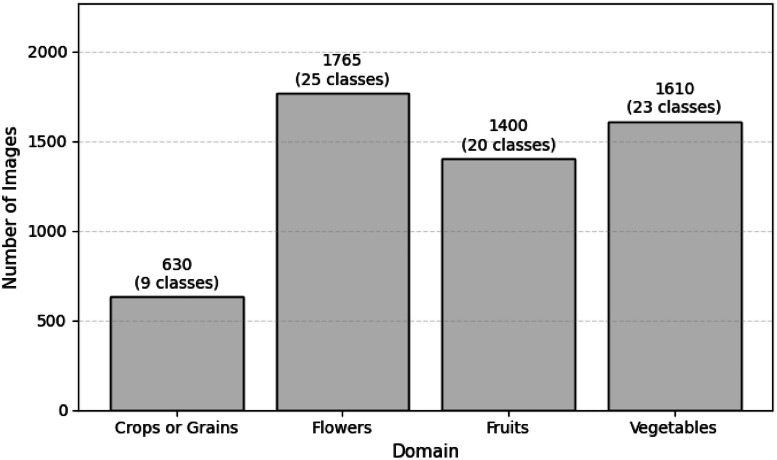
Image count distribution across four main classes.

**Fig. 2:**
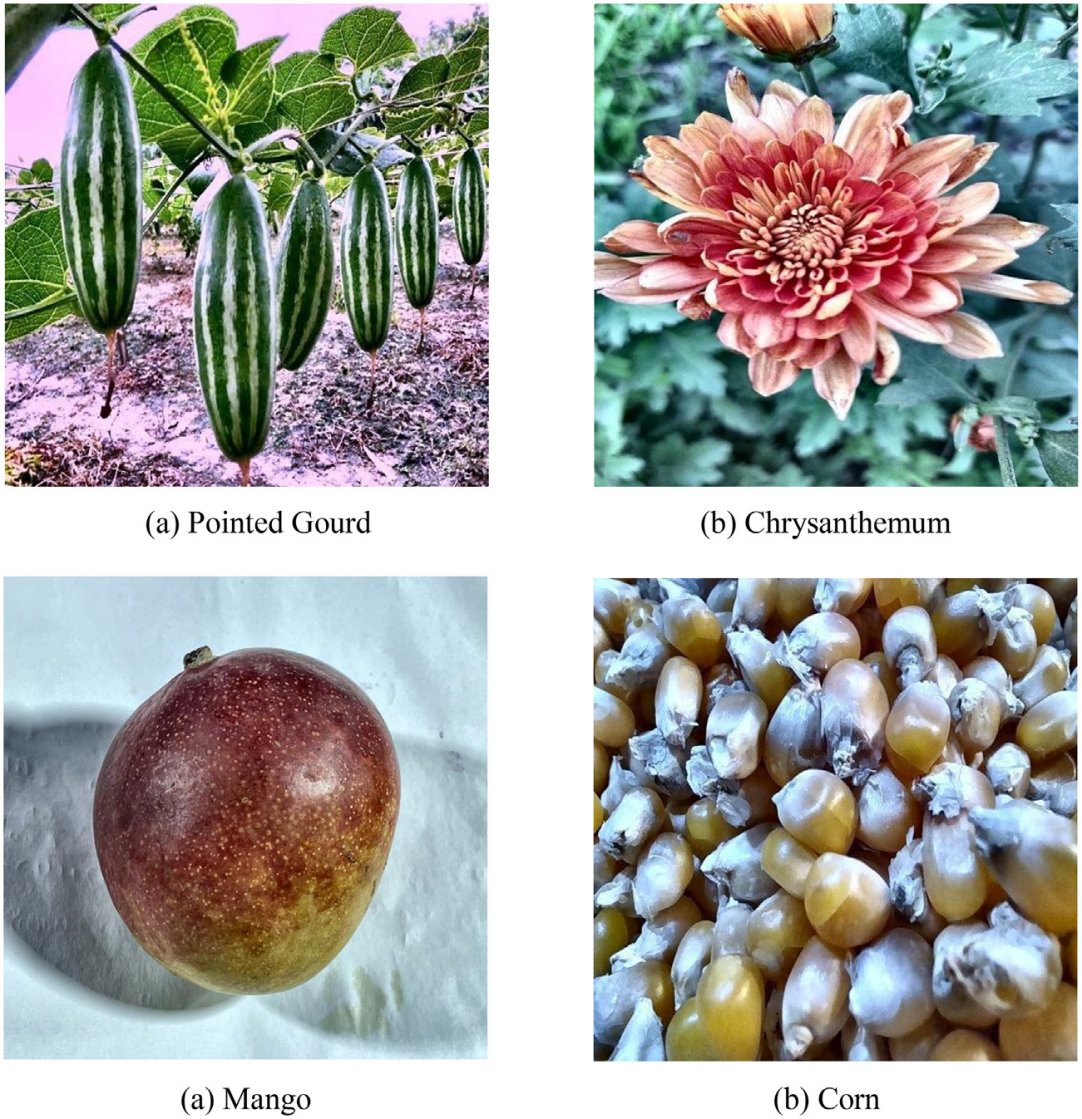
Sample images of vegetable, flower, fruit and corn/grain.

Fig. 1 illustrates the image distribution across domains, where Flowers contain the largest share with 1765 images, followed by Vegetables (1610), Fruits (1400), and Farming/Cereals (630). A detailed summary of the dataset composition is presented in Table 1, while representative sample images from each domain are shown in Fig. 2.

**Table 1:** Summary of the AMCD dataset composition.

Main Category	Number of Subclasses	Images (Count)	Image Type
Flowers	25	1765	JPG images
Vegetables	23	1610	JPG images
Fruits	20	1400	JPG images
Crops/Grains	9	630	JPG images
Total	77	5405	JPG images

## 4 Experimental Design, Materials and Methods

### 4.1 Data collection

Images were manually collected from local farms as well as from fresh markets and gardens within three districts of the Dhaka Division in Bangladesh (i.e., Savar, Dhamrai and Manikganj). Two different smart phones were used in order to take all images (Samsung Galaxy A21s [48 MP Primary sensor, 2 MP Macro] and iPhone 12 Pro Max [12 MP Wide camera]). All images were taken in natural light and during daytime throughout the range of 27 January 2025 to 20 May 2025 (a total of 108 days). Framing of images focused on centering the subject(s), having a reasonably clean background and varying viewpoint and included both single instance and group instance photographs for each category. No existing public datasets were used and all images are original field captures (Table 2).

**Table 2:** Comparison of AMCD with existing publicly available agricultural image datasets.

Dataset	Classes	Images	Multi-domain	Real-world Conditions	Region
Sara et al. [5]	7	∼3500	No	Partial	Bangladesh
PlantVillage [6]	38	54,306	No	No	Global
Fruits-360 [10]	90	61,934	No	No	Global
AMCD (Ours) [12]	77	5405	Yes	Yes	Bangladesh

### 4.2 Data preprocessing

**Fig. 3:**
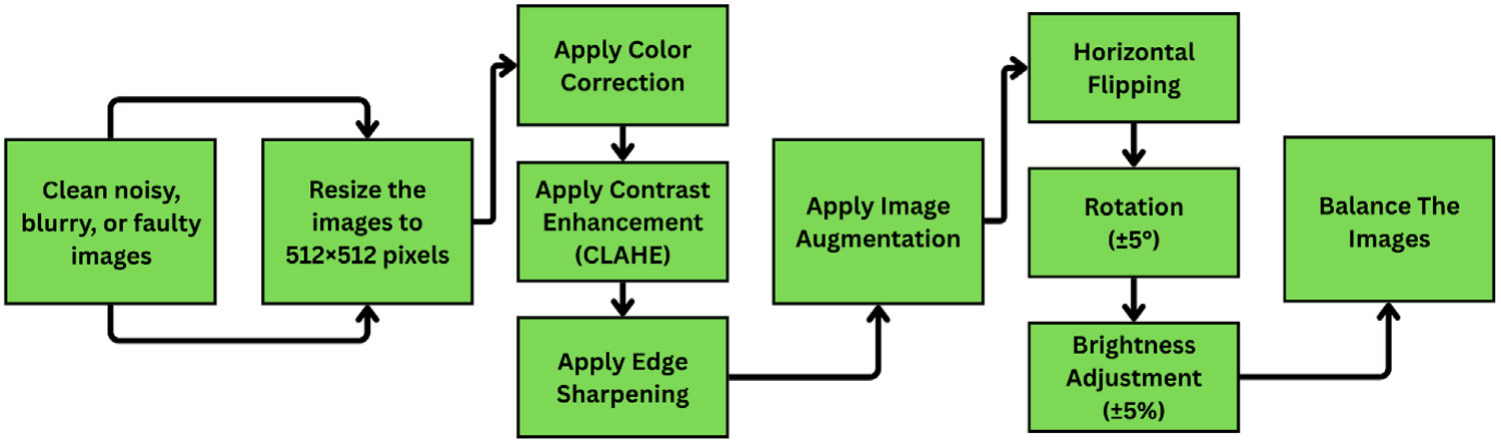
Image preprocessing workflow.

Sequentially processing and augmenting prepared data helped improve the overall quality of our datasets according to standards outlined in this section. The workflow is shown in Fig. 3.

**Image Cleaning:** Images had to be identified and eliminated due to poor quality factors (blurry images, fog and haze, poor focus, heavy obstruction, misaligned object placement) in order to provide quality images ultimately used for the dataset. This step ensures only discriminative, high quality samples enter the pipeline, reducing label noise

**Image Resizing:** All good images were resized to (512 × 512) pixels, which helped maintain a consistent dimension among retained images and therefore provided greater compatibility when using the images in typical deep learning development processes.

**Color Balancing and Correction:** Images that were poorly lit or had warm tone coloration were color balanced. The three components of color were adjusted (hue, saturation and brightness) to help retain accurate natural color against the items contained in the image and therefore provide greater accuracy to each item classified based on the color of that item. This corrects for the warm-tone bias introduced by smartphone sensors under direct sunlight, a common condition during field collection

**Enhancement of Contrast:** Local contrast and surface visibility were improved through the application of Contrast Limited Adaptive Histogram Equalization (CLAHE), which increased the local definition of textures as well as creating a better perception of fine textures such as grains and seeds. CLAHE was selected over global histogram equalization to avoid over-amplifying noise in uniform background regions.

**Enhancement of Edges:** The final processed images were sharpened prior to the final model building process to enhance the definition around object boundaries so that object features would be easier to differentiate during the model training phase. Sharpening was completed after climbing the contrast point due to the potential for excessive overshooting caused by previous processing. Sharpening was applied last to avoid compounding artifacts from earlier contrast enhancement steps.

### 4.3 Image augmentation

Non synthetic augmentation was used on subclasses with fewer than 70 original images to balance the data and obtain better generalization from the model. The augmentation methods used were: horizontal flipping, random rotation in each direction of ±5%, and adjusting brightness up or down by ±5%. Each of these operations produced controlled variations of the images relative to their orientation and illumination as well as ensured that the photos still looked like real agricultural photos, just as augmentation has previously been done for training the computer to recognize agricultural images using real photographs [13].

No synthetic images have been created (using generative adversarial networks (GANs) or diffusion models) for the project. All of the processing performed on the images can be found in Table 3.

**Table 3:** Image preprocessing and augmentation techniques applied in the AMCD dataset.

Processing Stage	Technique and Description
Image Cleaning	Removal of blurred, noisy, duplicate, or irrelevant images; selection of images with centered subject and proper focus.
Image Resizing	All images were resized to a uniform resolution of 512 × 512 pixels to ensure consistency and efficient model training.
Color Balance	Correction of dominant color casts caused by varying lighting conditions to improve visual consistency across the dataset.
Color Correction	Adjustment of hue, saturation, and brightness to better preserve natural appearance and real-world visual characteristics.
Contrast Enhancement	Contrast Limited Adaptive Histogram Equalization (CLAHE) was applied to improve local contrast and emphasize discriminative features.
Edge Sharpening	Image sharpening techniques were applied to enhance object boundaries and improve feature extraction capability.
Horizontal Flip (Aug)	Horizontal flipping was applied to introduce left-right orientation diversity and improve model generalization.
Rotation (Aug)	Small random rotations within approximately ±5° were applied to simulate natural camera angle variations.
Brightness Adjustment (Aug)	Minor brightness variations of approximately ±5% were applied to improve robustness against illumination changes.
Class Balancing Through Augmentation	Subclass image counts were balanced through non-synthetic augmentation. Most subclasses were standardized to 70 images per class, while the Dahlia subclass contained 85 original images.
Synthetic Data Usage	No synthetic images (e.g., GAN-generated, diffusion-generated, or simulated samples) were used. All images are real and field-collected.

### 4.4 Dataset splitting and baseline benchmark

The dataset was divided into training (80%), validation (10%), and test (10%) sets preserving class distribution across all subclasses. To evaluate the dataset's utility across architectures, four pretrained models were benchmarked under identical training conditions: EfficientNetB0, MobileNetV2, ResNet50, and VGG16. Each model was fine-tuned using the same two-phase transfer learning procedure (frozen backbone followed by top-30% fine-tuning), optimizer (Adam), loss function (categorical cross-entropy), and data split. Table 4 reports domain-level and subclass-level accuracy and macro-F1 on the held-out test set.

**Table 4:** Baseline classification performance of four pretrained models on AMCD.

Model	Domain Acc (%)	Domain F1 (%)	Subclass Acc (%)	Subclass F1 (%)	Params (M)
EfficientNetB0	99.07	99.21	99.44	99.25	4.15
MobileNetV2	98.33	98.41	99.81	99.80	2.36
ResNet50	99.63	99.68	99.63	99.71	23.75
VGG16	96.48	96.65	92.22	92.98	27.60

## Limitations

The AMCD dataset contains only healthy and non-diseased plant samples; therefore, it cannot be used directly for supervised plant disease detection, though it provides a necessary healthy-specimen baseline for comparative disease studies. Most subclasses were balanced to approximately 70 images using non-synthetic augmentation. Since augmented images are derived from originals present in the same dataset, random splitting may place augmented variants of the same source image in both training and test sets, which could inflate reported classification accuracy relative to a fully independent external evaluation.

Images were collected from three districts in the Dhaka Division of Bangladesh during January–May 2025 (dry season only), which excludes the monsoon and winter seasons during which the visual appearance of many tropical crops and flowers changes substantially. This limits representation across seasons, agroclimatic zones, and regional crop varieties beyond the Dhaka Division. The dataset does not include image-level GPS coordinates, timestamps, growth-stage labels, or device-specific identifiers. All images were standardized to 512 × 512 pixels, so original high-resolution visual details are not preserved. Baseline classification accuracy should therefore be interpreted with consideration of the dataset’s balanced and augmented structure.

## CRediT Author Statement

**Md Ahsan Karim:** Methodology, data augmentation, investigation, writing. **Md Tanjum An Tashrif:** Data curation, visualization, investigation. **Shahariar Hossain Mahir:** Data augmentation, visualization, software. **Md Kowsar Ahmed:** Writing, validation. **Umme Sara:** Conceptualization, review. **Mohammad Shorif Uddin:** Supervision, reviewing, editing. **Tufayel Haque Raha:** Data curation.

## Ethics Statement

This work does not involve human participants, animal experiments, or social media data. The authors followed the ethical requirements of Data in Brief during dataset collection and preparation.

## Data Availability

Mendeley DataAMCD: A Multi-Domain Agricultural Crop and Flower Image Dataset for Deep Learning-Based Classification (Original data).
